# Ultrathin Strut Biodegradable Polymer-Coated Sirolimus-Eluting Coronary Stents: Patient-Level Pooled Analysis From Two Indian Registries

**DOI:** 10.7759/cureus.41743

**Published:** 2023-07-11

**Authors:** Ramesh Babu Pothineni, Prakash Ajmera, Kamal Kumar Chawla, Sai Sudhakar Mantravadi, Abhijit Pathak, Manohar K Inamdar, Pankaj V Jariwala, Vikrant Vijan, Vinod Vijan, Anil Potdar

**Affiliations:** 1 Cardiology, Ramesh Hospitals, Vijayawada, IND; 2 Cardiology, Malla Reddy Narayana Multispeciality Hospital, Hyderabad, IND; 3 Cardiology, Gleneagles Global Hospital, Hyderabad, IND; 4 Cardiology, Swasthya Hospital and Medical Research Centre, Ahmednagar, IND; 5 Cardiology, Ashwini Hospital, Solapur, IND; 6 Cardiology, MaxCure Hospitals, Hyderabad, IND; 7 Cardiology, Vijan Cardiac and Critical Care Centre, Nashik, IND; 8 Cardiology, Parisoha Foundation Pvt. Ltd, Mumbai, IND

**Keywords:** percutaneous coronary intervention, multivessel disease, drug-eluting stents, diabetes, coronary occlusion

## Abstract

Background

Despite significant evolution in stent technology, female gender, and patients with diabetes mellitus, multivessel disease, total occlusions, long lesions, and small vessels represent the “Achilles' heel” of contemporary percutaneous coronary intervention (PCI). We performed a pooled analysis of high-risk subgroup on patient-level data from the T-Flex registry (1,203 patients) and a real-world Indian registry (1,269 patients), with the aim of assessing one-year safety and clinical performance of ultrathin strut biodegradable polymer-coated Supra family of sirolimus-eluting stents (SES) (Sahajanand Medical Technologies Limited, Surat, India) in the real-world, all-comer population.

Method

We pooled the following high-risk subgroups data from two all-comer registries: female gender (n=678), diabetes mellitus (n=852), multivessel disease (n=406), total occlusions (n=420), long lesions (≥28 mm) (n=1241), and small vessels (≤2.5 mm) (n=726). Both the registries included patients with coronary artery disease who underwent implantation of at least one SES belonging to the Supra family of stents from May 2016 until March 2018, irrespective of lesion complexity and comorbidities. The primary endpoint was the inci­dence of target lesion failure (TLF), a composite of cardiac death, target vessel myocardial infarction, and clinically indicated target lesion revas­cularization by percutaneous or surgical methods up to one year. The safety endpoint was stent thrombosis.

Results

According to prespecified high-risk subgroups, one-year rates of TLF and overall stent thrombosis, respectively, were as follows: female gender (4.9% and 0.6%), diabetes mellitus (6.9% and 1.0%), multivessel disease (6.4% and 0.8%), total occlusions (5.2% and 0.5%), long lesions (≥28 mm) (6.6% and 0.8%), and small vessels (≤2.5 mm) (6.1% and 1.3%).

Conclusion

This present pooled analysis demonstrated the one-year safety and clinical performance of ultrathin strut biodegradable polymer-coated Supra family of SES in a real-world, all-comer population, with considerably low rates of TLF and stent thrombosis.

## Introduction

Percutaneous coronary intervention (PCI) is the frontline treatment for interventional cardiologists in managing coronary artery disease (CAD). Although no universal definition of complex PCI exists [1], there is an emerging consensus that complex PCI and higher-risk indicated populations involve clinical risk factors and characteristics of coronary lesions and PCI procedures. Approximately 30% of all PCI procedures are considered complex depending on the lesion or anatomic factors [2]. The complex PCI population remains at higher risk of mid- to long-term adverse ischemic events [2, 3].

Coronary anatomic complexity serves as an important determinant in the decision-making between PCI and coronary artery bypass graft (CABG). U.S. Food and Drug Administration (USFDA) gave an on-label indication for the use of drug-eluting stents (DES) in simple, single, de novo coronary lesions in low-risk patients with CAD. Nevertheless, a number of PCIs are currently performed in patients with high-risk clinical and anatomic characteristics [4-8]. It has been reported that the complex PCI with DES is associated with a markedly higher ischemic risk, in a graded fashion, with expected lower procedural success rates with an increased risk of procedural complications [9, 10]. Several clinical trials have shown that DES has limited capability to prevent target lesion revascularization (TLR) in patients with complex lesions, whereas many studies confirmed similar clinical outcomes between patients with complex PCI and those with noncomplex PCI [11-13]. These conflicting findings have driven clinical studies to investigate the safety and effectiveness of the latest-generation DES in complex PCI subsets. 

The Supra family of stents (Sahajanand Medical Technologies Limited, Surat, India) are the latest-generation ultrathin strut biodegradable polymer-coated sirolimus-eluting stents (SES). One-year safety and efficacy of the Supra family of SES in real-world, all-comer CAD population has already been addressed in the literature [14-17]. However, there is a lack of data about its safety and efficacy in the most frequently encountered challenging PCI subsets - female gender and patients with diabetes mellitus, multivessel disease, total occlusions, long lesions, and small vessels. Against this background, we aimed to assess the one-year safety and clinical performance of the Supra family of SES in a real-world all-comer population, using a pooled analysis of high-risk subgroups on patient-level data from two all-comer registries [15, 17].

## Materials and methods

Study design and population

We pooled high-risk subgroup data from 2471 CAD patients enrolled in two all-comer registries: T-Flex registry (n=1,203) [15] and a real-world Indian registry (n=1,269) [17]. High-risk subgroups derived from two all-comer registries included female gender (n=678), diabetes mellitus (n=852), multivessel disease (n=406), total occlusions (n=420), long lesions (≥28 mm) (n=1241), and small vessels (≤2.5 mm) (n=726). Both the registries comprised patients with CAD who underwent implantation of at least one SES belonging to the Supra family of SES between May 2016 and March 2018, irrespective of lesion complexity and comorbidities. Institutional Ethics Committee (IEC) granted approval for the study protocol and both registries adhered to the tenets of the Declaration of Helsinki. Signed informed consent was obtained from each patient for data collection and analysis for research purposes and the use of anonymized clinical data.

Description of study stent

The study stents were ultrathin strut (60 µm) biodegradable polymer-coated SES designed with a unique long dual-Z link (LDZ-link) between ‘valley-to-valley’ on Tetrinium L-605 cobalt-chromium alloy as its stent platform (Sahajanand Medical Technologies Limited, Surat, India). These latest-generation stents feature a multilayer conformal coating on the surface, which contains 1.4 μg/mm2 of sirolimus in the centre and the innermost layer, amalgamate with a biodegradable polymeric matrix composed of a combination of hydrophobic [PLLA: poly(L-lactide)], PLCL: poly(L-lactide-co-caprolactone)], and outer drug-free hydrophilic polymers (PVP: polyvinylpyrrolidone). Approximately 80% of the drug is released within four weeks in biological media. The remaining drug is programmed to be released at a slow rate in about three months. Once the drug is released, biodegradable polymers progressively degrade into biologically acceptable molecules and excreted from the body. The average coating thickness of SES is between 4 and 6 μm.

Coronary interventional procedure and adjuvant medications

Coronary interventional procedures and adjuvant medica­tions were carried out in accordance with standard practice guidelines. All the patients were recommended for dual antiplatelet therapy (DAPT) for at least up to 12 months (aspirin 75-300 mg daily indefinitely and clopidogrel 75 mg daily or prasugrel 10 mg daily or ticagrelor 90 mg twice daily) after the procedure. Follow-up was obtained either via clinical follow-up or telephonic contact one year after stent implantation.

Clinical endpoint

Both the registries considered inci­dence of target lesion failure (TLF) as a primary endpoint, which was defined as a composite endpoint of cardiac death, target vessel myocardial infarction (TV-MI), and clinically indicated TLR (CI-TLR) by percutaneous or surgical methods at one-year. Events of stent thrombosis were assessed as an additional safety endpoint.

Statistical analysis

Data analysis was performed using the IBM Statistical Package for Social Sciences for Windows, version 20.0. (IBM Corp., Armonk, USA). Descriptive statistical analysis methods were used to perform data analysis; continuous variables were reported as mean ± standard deviation, and categorical variables were described as frequency and percentages. The TLF event curve was obtained using the Kaplan-Meier method.

## Results

The baseline demographic and clinical details of the pooled high-risk subgroups are outlined in Table 1.

**Table 1:** Demographic and clinical characteristics of high-risk subgroups CAD, Coronary artery disease; MI, Myocardial infarction; CABG, Coronary artery bypass graft; PCI, Percutaneous coronary intervention; STEMI, ST-elevation myocardial infarction; NSTEMI, non-ST-elevation myocardial infarction.

Characteristics	Female patients (n=678)	Diabetic mellitus (n=852)	Multivessel disease (n=406)	Total occlusion (n=420)	Long lesion (≥28 mm) (n=1241)	Small vessels (≤2.5 mm) (n=726)
Age (years), mean ± SD	57.7 ± 10.6	55.6 ± 9.8	58.01 ± 10.3	55.3 ± 10.8	56.0 ± 10.8	57.6 ± 10.0
Male, n (%)	-	575 (67.5)	288 (70.9)	302 (71.9)	929 (74.9)	491 (67.6)
Cardiovascular risk, n (%)						
Hypertension	362 (53.4)	566 (66.4)	193 (47.5)	189 (45.0)	578 (46.6)	340 (46.8)
Hypercholesterolemia	229 (33.8)	308 (36.2)	129 (31.8)	147 (35.0)	396 (31.9)	217 (29.9)
Diabetes mellitus	277 (40.9)	852 (100.0)	155 (38.2)	131 (31.2)	417 (33.6)	291 (40.1)
Smoking	86 (12.7)	123 (14.4)	55 (13.5)	79 (18.8)	232 (18.7)	107 (14.7)
Family history of CAD	15 (2.2)	31 (3.6)	6 (1.5)	14 (3.3)	198 (16.0)	24 (3.3)
Previous MI	54 (8.0)	75 (8.8)	28 (6.9)	36 (8.6)	102 (8.2)	60 (8.3)
Previous CABG	9 (1.3)	19 (2.2)	6 (1.5)	5 (1.2)	18 (1.5)	15 (2.1)
Previous PCI	43 (6.3)	74 (8.7)	21 (5.2)	30 (7.1)	91 (7.3)	66 (9.1)
Previous stroke	16 (2.4)	26 (3.1)	9 (2.2)	6 (1.4)	27 (2.2)	20 (2.8)
Renal insufficiency	5 (0.7)	13 (1.5)	5 (1.2)	7 (1.7)	18 (1.5)	16 (2.2)
Cardiogenic shock	19 (2.8)	21 (2.5)	10 (2.5)	8 (1.9)	32 (2.6)	22 (3.0)
Clinical presentation, n (%)						
Stable angina	183 (27.0)	192 (22.5)	77 (19.0)	66 (15.7)	250 (20.1)	133 (18.3)
Unstable angina	234 (34.5)	265 (31.1)	122 (30.0)	106 (25.2)	345 (27.8)	230 (31.7)
STEMI	180 (26.5)	167 (19.6)	94 (23.2)	141 (33.6)	334 (26.9)	175 (24.1)
NSTEMI	81 (11.9)	84 (9.9)	68 (16.7)	51 (12.1)	166 (13.4)	100 (13.8)

Briefly, the values of mean age and male predominance closely approached all subgroup populations. ST-elevation myocardial infarction (STEMI) was the most frequent clinical presentation in patients with total occlusions (33.6%). Stable angina (27.0%) and unstable angina (34.5%) were frequently evident in female patients. Non-ST-elevation myocardial infarction (NSTEMI) (16.7%) was the commonest in the multivessel disease subgroup.

The majority of the lesions endangered the left anterior descending artery (LAD) in all pooled high-risk subgroups. The number of stents deployed per patient for high-risk subgroups were as follows: female gender (1.31 ± 0.53), diabetic mellitus (1.3 ± 0.5), multivessel disease (1.96 ± 0.45), total occlusion (1.3 ± 0.5), long lesions (≥28 mm) (1.2 ± 0.4), and small vessels (≤2.5 mm) (1.1 ± 0.3). The remaining stent characteristics are elucidated in Table 2.

**Table 2:** Lesion characteristics of high-risk subgroups *American College of Cardiology (ACC)/American Heart Association (AHA) classification of coronary lesions. LAD, Left anterior descending artery; RCA, Right coronary artery; LCX, Left circumflex artery; SVG, Saphenous vein grafts.

Characteristics	Female patients	Diabetic mellitus	Multivessel disease	Total occlusions	Long lesions (≥28 mm)	Small vessels (≤2.5 mm)
No. of lesions	802	1024	824	436	1360	771
Disease vessel, n (%)						
Single-vessel disease	341 (50.3)	408 (47.9)	96 (23.6)	203 (48.3)	581 (46.8)	332 (45.7)
Double-vessel disease	264 (38.9)	348 (40.8)	261 (64.3)	178 (42.4)	519 (41.8)	319 (43.9)
Triple-vessel disease	73 (10.8)	96 (11.3)	49 (12.1)	39 (9.3)	141 (11.4)	75 (10.3)
Target-vessel location, n (%)						
LAD	391 (48.8)	487 (47.6)	326 (39.6)	192 (44.0)	684 (50.3)	397 (51.5)
RCA	266 (33.2)	313 (30.6)	265 (32.2)	175 (40.1)	470 (34.6)	145 (18.8)
LCX	142 (17.7)	215 (21.0)	229 (27.8)	67 (15.4)	205 (15.1)	224 (29.1)
Left main	1 (0.1)	2 (0.2)	4 (0.5)	2 (0.5)	0 (0.0)	1 (0.1)
SVG	2 (0.2)	7 (0.7)	0 (0.0)	0 (0.0)	1 (0.1)	4 (0.5)
Lesion details, n (%)						
Type A*	87 (10.8)	98 (9.6)	98 (11.9)	3 (0.7)	48 (3.5)	71 (9.2)
Type B1*	106 (13.2)	110 (10.7)	99 (12.0)	21 (4.8)	80 (5.9)	93 (12.1)
Type B2*	128 (16.0)	151 (14.7)	126 (15.3)	33 (7.6)	118 (8.7)	104 (13.5)
Type C*	481 (60.0)	665 (64.9)	501 (60.8)	379 (86.9)	1114 (81.9)	503 (65.2)
Total occlusion	97 (12.1)	136 (13.3)	71 (8.6)	436 (100.0)	243 (17.9)	122 (15.8)
Pre-dilatation	267 (33.3)	263 (25.7)	291 (35.3)	224 (51.4)	501 (36.8)	155 (20.1)
Post-dilatation	301 (37.5)	342 (33.4)	346 (42.0)	207 (47.5)	573 (42.1)	123 (16.0)
Stent details, n (%)						
Total no. of stents	891	1158	855	524	1441	807
No. of stents deployed per patient, mean ± SD	1.31 ± 0.53	1.3 ± 0.5	1.96 ± 0.45	1.3 ± 0.5	1.2 ± 0.4	1.1 ± 0.3
No. of stents deployed per lesion, mean ± SD	1.11 ± 0.34	1.1 ± 0.3	1.04±0.20	1.2 ± 0.4	1.1 ± 0.2	1.1 ± 0.4
Stent length (mm), mean ± SD	25.1 ± 8.9	25.5 ± 8.7	25.94±9.20	27.3 ± 9.1	34.0 ± 5.8	25.6 ± 9.2
Stent diameter (mm), mean ± SD	2.8 ± 0.3	2.8 ± 0.3	2.84 ± 0.30	2.9 ± 0.3	3.0 ± 0.3	2.5 ± 0.02
Stent length, n (%)						
8 mm	4 (0.4)	3 (0.3)	7 (0.8)	1 (0.2)	-	4 (0.5)
12 mm	60 (6.7)	83 (7.2)	58 (6.8)	21 (4.0)	-	71 (8.8)
16 mm	143 (16.0)	157 (13.6)	121 (14.2)	68 (13.0)	-	122 (15.1)
20 mm	171 (19.2)	217 (18.7)	149 (17.4)	78 (14.9)	-	121 (15.0)
24 mm	159 (17.8)	193 (16.7)	122 (14.3)	92 (17.6)	-	126 (15.6)
28 mm	107 (12.0)	159 (13.7)	128 (15.0)	68 (13.0)	447 (31.0)	116 (14.4)
32 mm	104 (11.7)	142 (12.3)	106 (12.4)	81 (15.5)	435 (30.2)	106 (13.1)
36 mm	54 (6.1)	99 (8.5)	67 (7.8)	43 (8.2)	244 (16.9)	50 (6.2)
40 mm	41 (4.6)	56 (4.8)	41 (4.8)	32 (6.1)	147 (10.2)	45 (5.6)
44 mm	23 (2.6)	24 (2.1)	34 (4.0)	19 (3.6)	95 (6.6)	26 (3.2)
48 mm	25 (2.8)	25 (2.2)	22 (2.6)	21 (4.0)	73 (5.1)	20 (2.5)
Stent diameter, n (%)						
2.25 mm	1 (0.1)	1 (0.1)	1 (0.1)	2 (0.4)	0 (0.0)	6 (0.7)
2.50 mm	263 (29.5)	324 (28.0)	245 (28.7)	130 (24.8)	363 (25.2)	801 (99.3)
2.75 mm	256 (28.7)	339 (29.3)	257 (30.1)	144 (27.5)	441 (30.6)	-
3.00 mm	269 (30.2)	353 (30.5)	252 (29.5)	184 (35.1)	462 (32.1)	-
3.50 mm	102 (11.4)	141 (12.2)	100 (11.7)	64 (12.2)	174 (12.1)	-
4.00 mm	0 (0.0)	0 (0.0)	0 (0.0)	0 (0.0)	1 (0.1)	-

The one-year follow-up data obtained for high-risk subgroups were as follows (Table 3): 96.6% in female gender, 94.7% in diabetic mellitus, 96.3% in multivessel disease, 96.9% in total occlusions, 95.5% in long lesions (≥28 mm), and 96.6% in small vessels (≤2.5 mm). One-year TLF rates in the predefined high-risk subgroups were: 4.9% in the female gender, 6.9% in diabetes mellitus, 6.4% in multivessel disease, 5.2% in total occlusions, 6.6% in long lesions (≥28 mm), and 6.1% in small vessels (≤2.5 mm). Overall stent thrombosis occurred in 0.6% of female patients, 1.0% in diabetes mellitus, 0.8% in multivessel disease, 0.5% in total occlusions, 0.8% in long lesions (≥28 mm), and 1.3% in small vessels (≤2.5 mm) high-risk subgroups.

**Table 3:** One-year clinical outcomes of high-risk subgroups TV-MI, Target vessel myocardial infarction; TLR, Target lesion revascularization; Non-TL TVR, Non-target lesion target vessel revascularisation.

Characteristics	Female patients (n=655)	Diabetic Mellitus (n=807)	Multivessel disease (n=391)	Total occlusion (n=407)	Long lesion (≥28 mm) (n=1185)	Small vessels (≤2.5 mm) (n=701)
All-cause death, n (%)	9 (1.4)	14 (1.7)	5 (1.3)	9 (2.2)	19 (1.6)	11 (1.6)
Cardiac death	5 (0.8)	6 (0.7)	3 (0.8)	5 (1.2)	11 (0.9)	5 (0.7)
Non-cardiac Death	4 (0.6)	8 (1.0)	2 (0.5)	4 (1.0)	8 (0.7)	6 (0.9)
TV-MI	15 (2.3)	19 (2.4)	9 (2.3)	7 (1.7)	36 (3.0)	20 (2.9)
TLR	12 (1.8)	31 (3.8)	13 (3.3)	9 (2.2)	31 (2.6)	18 (2.6)
Non-TL TVR	6 (0.9)	11 (1.4)	9 (2.3)	5 (1.2)	8 (0.7)	7 (1.0)
Overall stent thrombosis, n (%)	4 (0.6)	8 (1.0)	3 (0.8)	2 (0.5)	10 (0.8)	9 (1.3)
Target lesion failure, n (%)	32 (4.9)	56 (6.9)	25 (6.4)	21 (5.2)	78 (6.6)	43 (6.1)

**Figure 1:**
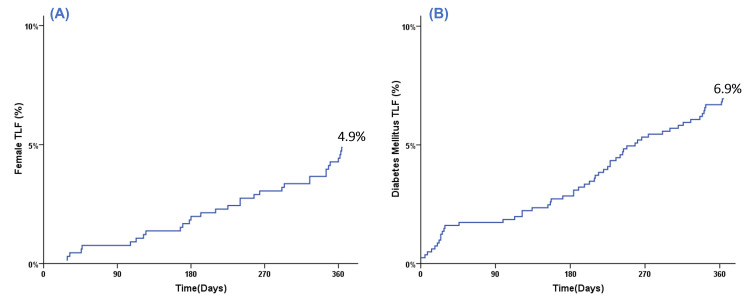
Kaplan-Meier event curve for cumulative target lesion failure (TLF) during one-year follow-up for (A) female patients and (B) diabetic mellitus

**Figure 2:**
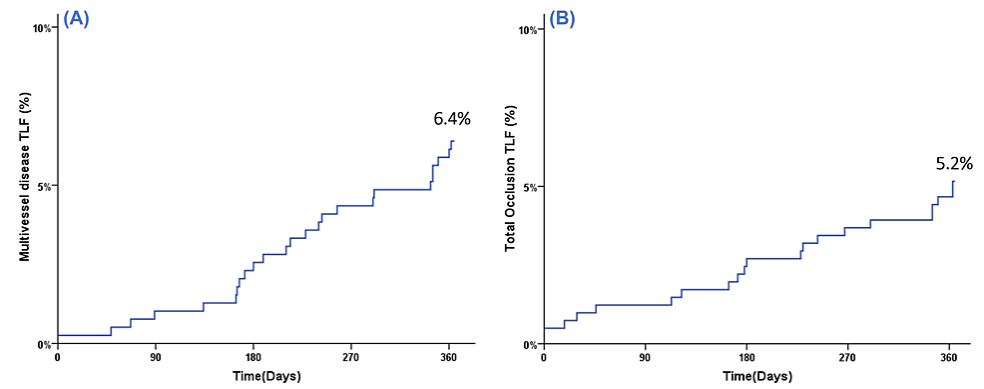
Kaplan-Meier event curve for cumulative target lesion failure (TLF) during one-year follow-up for (A) multivessel disease and (B) total occlusions

**Figure 3:**
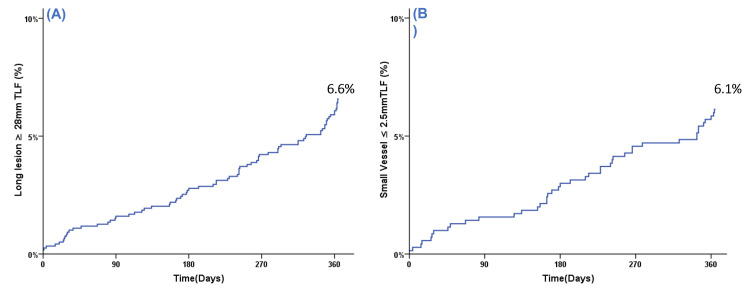
Kaplan-Meier event curve for cumulative target lesion failure (TLF) during one-year follow-up for (A) long lesions (≥28 mm) and (B) small vessels (≤2.5 mm)

One-year cumulative TLF rates for stated high-risk subgroups determined by the Kaplan-Meier method are depicted in Figures 1-3.

## Discussion

As expected, findings emerged from the present pooled analysis demonstrated that one-year safety and clinical performance of ultrathin strut (60 µm) biodegradable polymer-coated SES was favourable in treating complex PCI subsets, including female gender, diabetes mellitus, multivessel disease, total occlusions, long lesions, and small vessels, with low events of TLF and stent thrombosis.

Many previous studies asserted that gender disparities significantly affect the clinical outcomes of PCI. Compared to males, females are more prone to major cardiovascular adverse events (including death) and target vessel failure after PCI [18-22]. These higher event rates in females are mainly attributed to postmenopausal states, smaller coronary arteries [23], and smaller radial arteries [24]. Hormonal changes after menopause, as such, low plasma estrogen levels and elevated luteinizing hormone (LH) and follicle-stimulating hormone (FSH) levels, have a significant impact on the metabolism of plasma lipids and lipoproteins, ultimately leading to cardiac-related diseases [25, 26]. Smaller coronary artery diameter has proved to be associated with low PCI success rate and higher incidence of subsequent in‐hospital major adverse events [23]. Smaller radial arteries are more susceptible to vascular spasms, which is a primary cause of radial procedure failure [24]. Briefly, females designate one of the challenging subsets of the complex PCI population. Our study demonstrated a lower death rate (1.4%) in females than that of earlier reported rates in the Melbourne Interventional Group (MIG) PCI registry (6.4%) [27] as well as earlier published studies by Bugiardini et al. (10.5%) [28] and Otten et al. (5.3%) [29].

Diabetes is often viewed as a “dreadful threat” in patients with CAD. It has been allied with adverse events such as increased incidence of restenosis, repeat revascularization, stent thrombosis (ST), and all-cause mortality after PCI. Of concern, diabetes patients exhibit diverse anatomical patterns of CAD, including multivessel stenosis, smaller vessel size, left main involvement, and rapidly progressing, more diffuse, and longer atherosclerotic lesions [30], which make revascularization more challenging. A subgroup analysis of the SIRIUS trial [31] and the DIABETES trial [32] paved the way for beginning a new SES era in CAD patients with diabetes. However, safety-related concerns are raised with SES implantation as diabetic patients have increased thrombocyte aggregation and thrombotic events [33]. We found a 6.9% one-year TLF rate with ultrathin strut biodegradable polymer-coated SES in the diabetic population. This figure was numerically comparable with the values reported for Supraflex SES (5.8%) and Xience everolimus-eluting stents (EES) (Abbott Vascular, Santa Clara, California) (8.5%) in the TALENT trial [14], amphilimus SES (5.9%) and zotarolimus-eluting stents (ZES) (7.5%) in a study reported by Rozemeijer et al. [34], Orsiro SES (Biotronik, Bulach, Switzerland) from the BIOFLOW III Italian Satellite Registry (6.9%) [35], DES [i.e. ridaforolimus-eluting stents (RES) and ZES] from BIONICS trial (7.8%) [36], and Resolute Integrity ZES (Medtronic Inc., Santa Rosa, CA, USA) in the International Global Resolute Program (6.6%) [37] (Table 4).

**Table 4:** Summary of one-year target lesion failure rates for high-risk subgroups from the present study and previously reported studies SES, Sirolimus-eluting stent; EES, Everolimus-eluting stent; A-SES,Amphilimus SES; RES, Ridaforolimus-eluting stents; ZES,  Zotarolimus-eluting stent; CTO, Chronic total occlusions; SVD, Small vessel disease; SMT: Sahajanand Medical Technologies Limited

Trial name/Author	Study design	N	Stent	Manufacturer	TLF
Diabetic subgroup					
Present pooled analysis	-	852	Ultrathin-strut biodegradable polymer-coated SES	SMT	6.9%
Talent trial [14]	Prospective, randomised, single-blind, multicentre study	157 178	Supraflex SES; Xience EES	SMT; Abbott Vascular, Santa Clara, California	5.8% 8.5%
Rozemeijer et al. [34]	Single-center retrospective analysis	85 80	A-SES ZES	-	5.9% 7.5%
Bioflow-III Italian Satellite registry [35]	Nationwide, prospective, all-comer registry	192	Orsiro SES	Biotronik, Bulach, Switzerland	6.9%
International Global Resolute Program [37]	Pooled patient-level data for 5,130 patients from 5 controlled studies, with 1 randomized and 4 single-arm	878	Resolute Integrity ZES	Medtronic Inc., Santa Rosa, CA, USA	6.6%
Multivessel disease					
Present pooled analysis	-	406	Ultrathin-strut biodegradable polymer-coated SES	SMT	6.4%
e-Ultimaster registry [40]	Prospective, multicentre, observational registry	5,852	Ultimaster SES	Terumo Corporation, Tokyo, Japan	4.2%
Talent trial [14]	Prospective, randomised, single-blind, multicentre study	311	Supraflex SES; Xience EES	SMT; Abbott Vascular, Santa Clara, California	10% 5.7%
Total occlusions					
Present study	Pooled subgroup analysis of two real-world, all-comers, multicentre registries	420	Ultrathin-strut biodegradable polymer-coated SES	SMT	5.2%
e-Ultimaster registry [40]	Prospective, multicentre, observational registry	1774	Ultimaster SES	Terumo Corporation, Tokyo, Japan	3.3%
Bioflow-III Italian Satellite Registry [35]	Nationwide, prospective, all-comer registry	38	Orsiro SES	Biotronik, Bulach, Switzerland	5.3%
Long lesions					
Present pooled analysis	-	1241	Ultrathin-strut biodegradable polymer-coated SES	SMT	6.6%
Talent trial [14]	Prospective, randomised, single-blind, multicentre study (>18 mm lesion)	518 511	Supraflex SES; Xience EES	SMT; Abbott Vascular, Santa Clara, California	5.7% 7%
Sinha et al. [50]	Prospective, single-arm, investigator-initiated study (≥40 mm lesion)	684	Supralimus Grace SES	SMT	6.1%
e-Ultimaster registry [40]	Prospective, multicentre, observational registry (≥60 mm lesion)	3146	Ultimaster SES	Terumo Corporation, Tokyo, Japan	4.8%
Bioflow V trial [51]	Prospective, randomised, assessor blinded multicentre trial (>26 mm lesion)	78 36	Orsiro SES; Xience EES	Biotronik, Buelach, Switzerland; Abbott Vascular, Santa Clara, California	5% 17%
Spirit Prime Trial (long lesion registry) [54]	Prospective, non-randomized clinical trial (33- and 38-mm stents)	104	Xience Prime EES	Abbott Vascular, Santa Clara, CA	12.5%
Pooled Analysis from the SPIRIT and XIENCE V USA Prospective Multicenter trials [55]	Pooled analysis of Spirit II, III, IV, V, Spirit small vessel and XIENCE V USA (≥35 mm lesion)	323	Xience V EES	Abbott Vascular, Santa Clara, CA	8.9%
Small vessels					
Present pooled analysis	-	726	Ultrathin-strut biodegradable polymer-coated SES	SMT	6.1%
Talent trial [14]	Prospective, randomised, single-blind, multicentre study (≤2.75 mm vessel size)	518 511	Supraflex SES; Xience EES	SMT; Abbott Vascular, Santa Clara, California	8% 5.8%
Xience V USA Condition of Approval Post-Market study [57]	Single-arm, prospective multicenter study (2.5 mm stent size)	838	Xience V	Abbott Vascular, Santa Clara, CA	5.7%
Bioflow V trial [51]	Prospective, randomised, assessor blinded multicentre trial (≤2.75mm vessel size)	554 290	Orsiro SES; Xience EES	Biotronik, Buelach, Switzerland; Abbott Vascular, Santa Clara, CA	8% 11%
Century JSV study [58]	Prospective, multicenter, single-arm study (2.25 mm stent size)	70	Ultimaster SES	Terumo Corporation, Tokyo, Japan	4.3%
Spirit small vessel trial [59]	Prospective, single-arm, open-label study (<2.5 mm vessel size)	150	Xience V EES	Abbott Vascular, Santa Clara, CA	8.1%
Restore SVD China randomized trial [60]	Prospective, randomized, open-label, multicenter (≥2.0–<2.75 mm vessel size)	114	Resolute Integrity ZES	Medtronic Inc., Santa Rosa, CA, USA	5.0%

With a growing number of aging population with various comorbidities, including diabetes and obesity, the prevalence of multivessel disease has been projected to rise, representing 30% to 40% of CAD patients [38]. Patients with multivessel disease are more likely to have worse clinical outcomes than those with single‐vessel disease [39]. PCI and CABG are the established revascularization strategies in patients with multivessel CAD. Nonetheless, the selection of optimal revascularization strategies remains a subject of intense discussion and debate. The discovery of DES has greatly enriched the conventional PCI era with promising outcomes in complex lesions, including multivessel disease. Consistent with one-year TLF rate for multivessel disease with the Ultimaster SES (Terumo Corporation, Tokyo, Japan)** **in e-Ultimaster registry (4.2%) [40] and Supraflex SES (10%) and Xience EES (5.7%) in TALENT trial [14], the present study demonstrated 6.4% one-year TLF rate with ultrathin strut biodegradable polymer-coated SES in multivessel disease subgroup (Table 4).

Labeled as the most challenging and growing subset of the complex PCI population, total occlusions have been plagued by a number of procedural complications including coronary perforation, periprocedural myocardial infarction, arrhythmias, cardiogenic shock, stroke, major bleeding, donor vessel thrombosis, contrast-induced nephropathy, radiation damage, and death and adverse clinical events [41]. It has been stated that chronic total occlusions (CTO-PCI) are high-risk procedures with low success rates than standard PCI. A significant breakthrough in recanalization techniques, the introduction of dedicated equipment and the development of systematic algorithmic approaches have significantly improved procedural success and reduced the occurrence of major complications [42, 43]. Numerous risk factors including sex, hypertension, smoking, and diabetes can affect the prognosis in patients with CTO lesions, which could lead to an increase in the frequency of adverse cardiovascular events. Control of these influential risk factors should be encouraged with routine follow-up. Despite the presence of multiple comorbidities/history, such as hypertension (45.0%), hypercholesteremia (35.0%), diabetes mellitus (31.2%), and previous revascularization (1.2% CABG and 7.1% PCI), we observed a 5.2% one-year TLF rate with ultrathin strut biodegradable polymer-coated SES in total occlusions subgroup. These values were in accordance with the values reported for Ultimaster SES in the e-Ultimaster registry (3.3%) [40], and Orsiro SES in BIOFLOW III Italian Satellite Registry (5.3%) [35] (Table 4).

Long lesions are associated with more adverse events after DES implantation, including higher rates of restenosis, periprocedural myocardial infarction, and stent thrombosis, which portrays the Achilles' heel of PCI. Despite refinement in devices and techniques, the intervention of long lesions poses technical difficulties such as stent deliverability, stent overlap, geographical miss, and prolonged intracoronary manipulation because of the multiple and overlapping stent placement which may impair the vessel wall integrity [44]. Mounting evidence supports the concept that multiple stent implantations in long lesions appear to have similar clinical outcomes to that of single long stent implantation [45-48]. The implantation of a single long stent yield reduction in catheterization time, dye volume for the patient, radiation exposure for both patient and operator, and procedural costs, compared to multiple stent implantations [48]. Long lesions have been frequently under-represented in clinical trials because of apparent heterogeneity in published definitions and limited stent length [49]. Latest-generations DES have emerged to treat long lesions in concert with unique stent characteristics, such as low loss rate to reduce restenosis risks, thin struts to enhance deliverability and reduce the risk of periprocedural infarction, and availability of long lengths to minimize overlap and avoid geographical miss [44].

The present study highlighted the safety and efficacy of a single ultrathin-strut biodegradable polymer-coated SES in treating long lesions (≥28 mm), with an acceptably low TLF rate of 6.6% at one year. This figure was numerically compared with one-year TLF rate documented with long lesions treated with Supraflex SES (5.7%) and Xience EES (7%) in TALENT trial [14], Supralimus Grace SES in a study reported by Sinha et al. (6.1%) [50], Ultimaster SES in e-Ultimaster registry (4.8%) [40], Orsiro SES (5%) and Xience EES (17%) in BIOFLOW V trial [51], Xience EES family (Xience EES and Xpedition EES) (2.6%) in the study reported by Sim et al. [52] and DES (SES and EES) (2.9%) in the study reported by Rajesh et al. [53], Xience Prime EES in the SPIRIT PRIME study (12.5%) [54], Xience V EES (8.9%) in a pooled analysis from the SPIRIT and XIENCE V USA prospective multicenter trials [55] (Table 4).

Although the history of PCI has been transitioned from plain old balloon angioplasty and bare metal stents to a variety of DES generations, clinical outcomes in small vessels remain far from satisfactory than those with large vessels. Higher incidences of in-stent restenosis and overall recurrent ischemic events have been reported after PCI of small vessels, which mainly occurs due to neointimal hyperplasia and inflammation [56]. There is a lack of standardized definitions for small vessel CAD. At one year, the TLF occurred in 6.1% of patients with ultrathin strut biodegradable polymer-coated SES in our subgroup of small vessels (≤2.5 mm reference vessel diameter). This TLF rate compared favourably with previous data wherein small lesions treated with Supraflex SES (8%) and Xience EES (5.8%) in the TALENT trial [14], Xience V EES from Xience V USA Condition of Approval Post-Market study (5.7%) [57], Orsiro SES (8%) and Xience EES (11%) from BIOFLOW V trial [51], Ultimaster SES in CENTURY JSV study (4.3%,) [58] Xience V EES in SPIRIT Small Vessel Trial (8.1%) [59], and Resolute Integrity ZES in the RESTORE SVD China Randomized Trial (5.0%) [60] (Table 4).

The rates of stent thrombosis in all six subgroups were also comparable with other similar studies [36, 41, 52] - 0.6% in female patients, 1% in patients with diabetes mellitus, 0.8% in patients with multi-vessel disease, 0.5% in patients with total occlusion, 0.8% in patients with long coronary lesions (≥28 mm), and 1.3% in patients with small diameter vessels (≤2.5 mm).

The key strength of this pooled subgroup analysis is that we focused exclusively on complex-PCI populations from two large-scale, multicentre, real-world Indian registries with adequate patient sizes in each subgroup; hence, the results can be generalized to the overall population.

Thankful to the latest-generation DES, which has appreciably improved procedural success rates in complex PCI. Overall, the results with the ultrathin strut SES in this pooled analysis were favourable in terms of low rates of TLF rate and stent thrombosis at one year. The Supra family of SES demonstrated favourable clinical outcomes in challenging lesion subsets and patient population, which is mainly attributable to stent design innovation, including ultrathin strut (60 µm) along with biodegradable polymer matrix and unique LDZ-link design.

## Conclusions

This pooled subgroup analysis confirmed the one-year safety and clinical performance of ultrathin strut biodegradable polymer-coated Supra family of SES in a real-world, all-comer Indian population, with markedly low rates of TLF and stent thrombosis.

The Supra family of SES has already been established as highly deliverable drug-eluting stents with low clinical event rates. This analysis covered an extensive range of complex and heterogenous PCI population in real-world settings providing additional data on efficacy and safety in complex patient and lesion subsets.
